# Plasma trimethylamine N-oxide (TMAO): associations with cognition, neuroimaging, and dementia

**DOI:** 10.1186/s13195-024-01480-1

**Published:** 2024-05-20

**Authors:** Amber Yaqub, Dina Vojinovic, Meike W. Vernooij, P. Eline Slagboom, Mohsen Ghanbari, Marian Beekman, Jeroen van der Grond, Thomas Hankemeier, Cornelia M. van Duijn, M. Arfan Ikram, Shahzad Ahmad

**Affiliations:** 1 Department of Epidemiology, Erasmus MC, University Medical Center, PO Box 2040, Rotterdam, CA 3000 the Netherlands; 2 Section of Molecular Epidemiology, Department of Biomedical Data Sciences, Leiden University Medical Center, Leiden, The Netherlands; 3 Department of Radiology and Nuclear Medicine, Erasmus MC, University Medical Center, Rotterdam, the Netherlands; 4 Department of Radiology, Leiden University Medical Center, Leiden, the Netherlands; 5 Division of Analytical Biosciences, Leiden Academic Centre for Drug Research, Leiden University, Leiden, Netherlands; 6 Nuffield Department of Population Health, University of Oxford, Oxford, UK

**Keywords:** Gut-microbiome, Metabolites, Cognition, Neuroimaging, Dementia, Population-based

## Abstract

**Background:**

The gut-derived metabolite Trimethylamine N-oxide (TMAO) and its precursors - betaine, carnitine, choline, and deoxycarnitine – have been associated with an increased risk of cardiovascular disease, but their relation to cognition, neuroimaging markers, and dementia remains uncertain.

**Methods:**

In the population-based Rotterdam Study, we used multivariable regression models to study the associations between plasma TMAO, its precursors, and cognition in 3,143 participants. Subsequently, we examined their link to structural brain MRI markers in 2,047 participants, with a partial validation in the Leiden Longevity Study (*n* = 318). Among 2,517 participants, we assessed the risk of incident dementia using multivariable Cox proportional hazard models. Following this, we stratified the longitudinal associations by medication use and sex, after which we conducted a sensitivity analysis for individuals with impaired renal function.

**Results:**

Overall, plasma TMAO was not associated with cognition, neuroimaging markers or incident dementia. Instead, higher plasma choline was significantly associated with poor cognition (adjusted mean difference: -0.170 [95% confidence interval (CI) -0.297;-0.043]), brain atrophy and more markers of cerebral small vessel disease, such as white matter hyperintensity volume (0.237 [95% CI: 0.076;0.397]). By contrast, higher carnitine concurred with lower white matter hyperintensity volume (-0.177 [95% CI: -0.343;-0.010]). Only among individuals with impaired renal function, TMAO appeared to increase risk of dementia (hazard ratio (HR): 1.73 [95% CI: 1.16;2.60]). No notable differences were observed in stratified analyses.

**Conclusions:**

Plasma choline, as opposed to TMAO, was found to be associated with cognitive decline, brain atrophy, and markers of cerebral small vessel disease. These findings illustrate the complexity of relationships between TMAO and its precursors, and emphasize the need for concurrent study to elucidate gut-brain mechanisms.

**Supplementary Information:**

The online version contains supplementary material available at 10.1186/s13195-024-01480-1.

## Background

Dementia is a leading cause of disability and dependency among older adults, with approximately 10 million new diagnoses per year worldwide [1]. In the absence of definitive curative treatments, there has been a growing interest in identifying potentially modifiable risk factors [2]. The microbiome has emerged as an important target for prevention, with studies indicating that its composition and diversity [3], as well as specific bacterial strains and their metabolites [4], may impact the risk of cognitive decline and dementia. This is thought to be mediated by the gut-brain axis, a two-way communication system between the gastrointestinal tract and the central nervous system. Recently, the diet-derived Trimethylamine N-oxide (TMAO) has gained considerable attention due to its potential role in promoting inflammation and atherosclerosis, factors that are both implicated in the pathophysiology of dementia [5].

Although small amounts of TMAO can be ingested directly via consumption of fish, most of it is produced by gut-microbiota of the small intestine from its precursors including choline, betaine, carnitine, and deoxycarnitine, which are found in meat, eggs and dairy [6]. These precursors are first metabolized into trimethylamine (TMA), which is then oxidized by hepatic flavin-containing monooxygenase 3 (FMO3) enzymes to TMAO. A growing body of evidence suggests a link between TMAO and hallmarks of vascular disease [6], such as platelet hyperreactivity [7] and endothelial dysfunction [8]. In addition, studies in mice have shown that TMAO can induce neuronal senescence, aggravate oxidative damage, promote neuroinflammation and impair mTOR signalling [9], all of which increase the susceptibility for cognitive impairment. TMAO is also capable of crossing the blood-brain barrier, where its levels in the cerebrospinal fluid were previously linked to biomarkers of Alzheimer’s disease [10], the most common form of dementia. Among patients with atrial fibrillation [11] and stroke [12], who generally are at increased risk of dementia, TMAO has been associated with markers of cerebral small vessel disease, such as white matter hyperintensities. Thus far, evidence on the detrimental effects of TMAO on the brain largely derives from animal studies or small-scale clinical samples [13], whilst population-based evidence on the association between TMAO and dementia is limited. By studying TMAO along with its precursors, we may gain additional insights into their respective roles in the process of neurodegeneration. Therefore, in this population-based study, we explored whether TMAO and its precursors are associated with markers of neurodegeneration, such as a decline in general cognition, structural imaging markers of brain atrophy or cerebral vessel disease and eventually, incident dementia. Hereby, we aim to elucidate the mechanisms by which TMAO and its precursors are involved in the bidirectional communication between the gut and the brain.

## Methods

### Study cohorts

This study was embedded in the Rotterdam Study (RS), with a partial validation for neuroimaging associations in the Leiden Longevity Study (LLS). A brief description of both cohorts can be found in Methods S1 [14, 15]. Both studies comply with Declaration of Helsinki and are approved by the Medical Ethical Committee of the respective institutional review boards (registration number MEC 02.1015 and P01.113). All participants provided written informed consent.

The current study includes participants of the RS for whom plasma levels of TMAO and its precursors were measured at study entry (*n* = 3,933), originating from the fourth examination round (2002–2005) of the first subcohort (RS-I-4) and the second examination round (2012–2014) of the third subcohort (RS-III-2). Out of these eligible participants, we identified participants who were dementia-free at baseline and subsequently created subsamples of participants who had (1) complete data on neuropsychological tests (*n* = 3,143) or (2) a brain MRI during the same center visit (*n* = 2,047) or (3) follow-up for incident dementia (*n* = 2,517). For validation of neuroimaging associations in the LLS, we only included offspring or long-lived sibling-pairs and partners of the offspring, who were dementia-free and had data on plasma levels of TMAO and its precursors, as well as brain MRI (*n* = 318).

### 2.2 Assessment of TMAO and its precursors in plasma

Plasma samples were obtained from fasting participants of the RS and non-fasting participants of the LLS using ethylenediaminetetetacetic acid tubes. TMAO and its precursors were quantified by mass spectrometry in a similar manner for the RS and LLS. An extensive description of the analytical techniques can be found in Methods S2 [16].

### Assessment of general cognition

Between 2002 and 2005, the protocol of the Rotterdam Study was extended with a comprehensive neuropsychological test battery for cognitive assessments. Three Stroop tests (reading, color naming and interference tasks), a letter-digit substitution task (LDST), a categorical Word Fluency Test (WFT), a Purdue pegboard (PPB) tests for the left hand, right hand and both hands and a 15-word verbal learning test based on Rey’s recall of words (15-WLT) were added to the protocol. As described in our previous publications [17], a summary measure of general cognition (‘G-factor’) was created using the first component of the principal component analysis that included the delayed recall score of the Stroop interference test, LDST, verbal fluency task, the PPB test and the 15-WLT. The variance in cognitive test scores explained by this G-factor was 50.3% for RS I-4 and 51.9% for RS III-2.

### Neuroimaging protocol

MRI scans of the brain, including T1-weighted, FLAIR, and proton density–weighted sequences, were available in both the RS and the LLS studies for the purpose of evaluating brain volumetry, including total brain, gray matter, white matter, hippocampus, and white matter hyperintensities. Participants with large strokes hampering segmentation results were excluded. Diffusion tensor imaging was used for determining the mean diffusivity and fractional anisotropy. Additional information on preprocessing steps, segmentation methods and other technical details is presented in Methods S3 [18–31].

### Dementia screening and surveillance

The ascertainment methods for dementia in the RS have been described in full in our previous publications [32]. Participants were cognitively screened using the Mini-Mental State Examination (MMSE) and the Geriatric Mental State (GMS) Schedule organic level, both at baseline and subsequent center visits. Participants scoring less than < 26 for MMSE or > 0 for GMS underwent further investigation and informant interview, including the Cambridge Examination for Mental Disorders of the Elderly. Data acquired from in-person screening was supported by data from the electronic linkage of the study database with medical records from general practitioners and the regional institute for outpatient mental health care, which facilitated continuous monitoring for dementia or cognitive disturbances between center visits.

The final diagnosis was established via a consensus panel of study physicians and was led by a consultant neurologist, who adhered to standard criteria for dementia (DSM-III-R) and Alzheimer’s disease (NINCDS-ADRDA) to determine presence, probability, and subtype of dementia. Follow-up was complete until January 1st 2018, and participants were censored within this follow-up period at date of dementia diagnosis, date of death, date of loss to follow-up, or January 1st 2018, whichever came first. Only participants from RS-I-4 were included for this analysis, as the number of individuals with incident dementia in the younger RS-III-1 sample was limited.

### Covariables

Assessment of covariables (educational attainment, smoking status, history of stroke, history of coronary heart disease (CHD), medication use, blood pressure, body mass index, total cholesterol, HDL-cholesterol, diabetes, e.g.) was comparable between the RS and LLS, and detailed further in Methods S4.

### Statistical analysis

#### Associations of TMAO and its precursors with cognition and brain MRI

Prior to analysis, plasma levels of TMAO and its precursors (in µmol/l) were log transformed to achieve normal distributions. The volume of white matter hyperintensities (cm^3^) was also log-transformed, as it had a left-skewed distribution. All structural imaging markers on brain MRI (cm^3^) were standardized to facilitate comparison. Cross-sectional associations between plasma levels of TMAO and its precursors with general cognition (G-factor) as well as brain MRI markers, were explored with linear regression models. All effect estimates are presented as mean differences, reflecting a log-unit change in the metabolite on the outcome, and its corresponding 95% confidence intervals (95% CIs). Due to slight variations in the assessment among samples, effect estimates were pooled using a random-effects meta-analysis using the inverse variance method and the DerSimonian-Laird estimator [33], with P-_Het_ that represents the P-value for heterogeneity. The pooled estimates were also summarized in cluster plots to display how plasma levels of TMAO and its precursors vary across cognition levels and how they associate with various neuroimaging markers.

For all statistical models, the selection for potential confounders was guided by literature review and biological plausibility [34]. Model I was adjusted for the main potential confounders, including age, sex, education, use of lipid lowering medication and body mass index. Model II was additionally adjusted for total cholesterol, HDL-cholesterol, smoking, hypertension and history of coronary heart disease, which are likely confounders, but could also be mediators. For all models that included structural imaging markers on brain MRI, we additionally adjusted for intracranial volume and, if applicable, the time-interval between blood sampling and MRI. Models pertaining to white matter hyperintensities, fractional anisotropy and mean diffusivity were additionally adjusted for (normal appearing) white matter volume. Only participants with complete data on covariables were included.

#### Associations of TMAO and its precursors with incident dementia

Relationships between plasma levels of TMAO and its precursors with incident dementia and Alzheimer’s disease, were determined for participants of RS-I-4 using Cox proportional hazard models, from which we obtained hazard ratios (HRs) and 95% CIs. Models were adjusted for a similar set of potential confounders as described earlier.

#### Stratified, sensitivity and supplementary analyses

Plasma levels of TMAO and its precursors may be affected by use of antibiotics and proton pump inhibitors (PPIs), as well as impaired clearance by the kidneys. They can also differ by sex. This information was only available for a subset of participants followed for incident dementia in the RS. In this subset, we performed a stratified analysis, where we studied associations of TMAO and its precursors with incident dementia, separately for non-users and users of antibiotics or PPIs. As a sensitivity analysis, we studied associations between TMAO and its precursors with incident dementia, among those with impaired renal function (estimated glomerular filtration rate < 60 mL/min). We also studied whether associations differed by sex. In a supplementary analysis, we assessed the correlation between TMAO and its precursors using Pearsons’ correlation coefficients.

#### Significance thresholds and software used

Multiple testing correction was applied using the false discovery rate (FDR) of Benjamini-Hochberg [35], and a suggestive association was considered at a nominal significance threshold of α = 0.05 (*P* ≤ 0.05). Analyses were performed using R version 3.6.1 (packages tidyr, dplyr, lubridate, foreign, and survival) and IBM SPSS Statistics version 24.0 (IBM Corp, Somers, NY), after which the meta-analysis was performed using the R package meta.

## Results

Of 3,933 participants of the RS with data on plasma levels of TMAO and its precursors, 3,143 participants had complete data on general cognition, 2,047 participants visited the center for a brain MRI after blood sampling and 2,517 participants were followed for incident dementia (Figure S1). In addition, a total of 318 participants of the LLS with data on plasma levels of TMAO and its precursors and brain MRI was included to validate the associations with brain MRI. Characteristics of each study sample are presented in Table 1. The mean age of study participants ranged from 59.3 years in the LLS (standard deviation (SD), ± 6.6) to 75.0 years (SD ± 6.0) in the RS, with slightly less females present in the LLS sample (53.0%) compared to RS (56.9%). Along with being older, participants in the RS had a different distribution of educational attainment than the LLS sample and more cardiovascular comorbidity.

**Table 1:** Baseline characteristics of study participants

Study	Rotterdam Study	Rotterdam Study	Rotterdam Study	Leiden Longevity Study	Rotterdam Study
Characteristic	Total sample (*N* = 3933)	Subsample with data on cognition (*N* = 3143)	Subsample with brain MRI (*N* = 2047)	Subsample with brain MRI (*N*=318)	Subsample followed for incident dementia (*N* = 2517)
Sex (%)					
Female	2278 (57.9)	1817 (57.8)	1164 (56.9)	169 (53.0)	1457 (57.9)
Age in years (mean, SD)	70.7 (8.4)	70.3 (8.1)	67.17 (7.3)	59.3 (6.6)	75.0 (6.0)
Cohort (%)					
RS-I-4	2557 (65.0)	2024 (64.4)	947 (46.3)	-	2517 (64.9)
RS-III-2	1376 (35.0)	1119 (35.6)	1100 (53.7)	-	0 (0.0)
Educational attainment (%)					
Primary education	463 (11.8)	326 (10.4)	169 (8.3)	127 (40.0)	347 (13.9)
Further education	2717 (69.5)	2201 (70.5)	1383 (67.9)	78 (24.4)	1849 (74.0)
Higher education	730 (18.7)	597 ( 19.1)	485 (23.8)	113 (35.6)	303 (12.1)
Body mass index in kg/m^2^ (mean, SD)	27.4 (4.3)	27.4 (4.2)	27.2 (4.1)	25.4 (3.4)	27.4 (4.1)
Smoking status (%)					
Never	1208 (31.2)	961 (31.1)	642 (31.8)	111 (35.8)	742 (30.2)
Former	2211 (57.2)	1770 (57.3)	1145 (56.7)	172 (54.1)	1451 (59.0)
Current	448 (11.6)	359 (11.6)	234 (11.6)	35 (11.1)	266 (10.8)
Hypertension (%)	2117 (54.1)	1639 (52.4)	954 (46.8)	55 (17.1)	1452 (58.0)
Total cholesterol (mmol/L)	5.6 (1.0)	5.6 (1.0)	5.6 (1.0)	5.6 (1.2)	5.6 (1.0)
HDL cholesterol (mmol/L)	1.5 (0.4)	1.5 (0.4)	1.5 (0.4)	1.4 (0.4)	1.4 (0.4)
Use of lipid lowering drugs	960 (24.5)	743 (23.7)	504 (24.7)	50 (15.7)	579 (23.1)
*APOE*-ε4 carrier (%)	1017 (27.5)	829 (28.0)	544 (28.2)	85 (26.8)	637 (26.6)
Diabetes (%)	297 (7.6)	223 (7.1)	123 (6.0)	9 (2.8)	212 (8.4)
History of stroke (%)	153 (3.9)	0 (0.0)*	0 (0.0)*	3 (1.0)	122 (4.8)
History of CHD (%)	349 (9.0)	267 (8.6)	126 (6.2)	3 (1.0)	283 (11.5)
		**Plasma levels of gut-related metabolites in µmol/l**			
Betaine (mean, SD)	34.8 (10.2)	34.8 (10.0)	35.1 (10.0)	38.0 (12.6)	34.3 (10.0)
Carnitine (mean, SD)	43.5 (8.7)	43.5 (8.7)	43.8 (8.6)	41.3 (9.2)	43.0 (8.5)
Choline (mean, SD)	9.6 (2.3)	9.6 (2.3)	9.3 (2.1)	10.9 (2.6)	9.9 (2.3)
Deoxycarnitine (mean, SD)	0.9 (0.2)	0.9 (0.2)	0.9 (0.2)	0.8 (0.2)	0.9 (0.2)
TMAO (mean, SD)	6.6 (9.2)	6.5 (9.5)	6.4 (9.4)	4.8 (5.5)	6.8 (8.9)

*Note* Unless specified otherwise, mean values and SD are displayed for continuous measures and absolute numbers are presented for categorical values

*Abbreviations* N = number of participants included in the study, SD = standard deviation, TMAO = Trimethylamine N-oxide. Plasma levels of gut-related metabolites are presented in µmol/l

*Participants with a history of stroke or dementia were excluded from the subsample

### Associations between plasma levels of TMAO, its precursors and cognition (G-factor)

As presented in Table 2, higher levels of TMAO were related to lower cognition, but this did not reach statistical significance. Instead, higher levels of choline were significantly associated with lower cognition (adjusted mean difference in model I: -0.170 [95% confidence interval (CI) -0.297;-0.043], *P* = 0.009, P-_Het_=0.329). Additional adjustment for potential confounders did not alter this association for choline (model II: -0.181 [95% CI: -0.323;-0.038], *P* = 0.013, P-_Het_= 0.270) and similar conclusions were drawn from the hierarchical cluster plot across different levels of cognitive functioning. (Figure S2).

**Table 2:** Associations between plasma levels of TMAO, its precursors and cognition (G-factor)

G-factor							
	RS I-4 (*N* = 1984)		RS III-2 (*N* = 1116)		Meta-analysis (*N* = 3100)		
Model I	Mean difference (95% CI)	P-value	Mean difference (95% CI)	P-value	Mean difference (95% CI)	P-value	P-_Het_
Betaine	0.127 (-0.017 ; 0.271)	0.084	-0.036 (-0.190 ; 0.118)	0.647	0.048 (-0.112 ; 0.208)	0.557	0.132
Carnitine	0.032 (-0.156 ; 0.219)	0.740	-0.044 (-0.242 ; 0.154)	0.666	-0.004 (-0.140 ; 0.132)	0.953	0.585
Choline	-0.112 (-0.286 ; 0.061)	0.203	**-0.239 (-0.427 ; -0.050)**	**0.013**	**-0.170 (-0.297 ; -0.043)**	0.009*	0.329
Deoxycarnitine	0.037 (-0.120 ; 0.193)	0.644	-0.035 (-0.258 ; 0.188)	0.756	0.013 (-0.115 ; 0.142)	0.840	0.605
TMAO	0.001 (-0.055 ; 0.057)	0.970	-0.036 (-0.095 ; 0.023)	0.229	-0.017 (-0.058 ; 0.024)	0.418	0.375
	RS I-4 (*N* = 1912)		RS III-2 (*N* = 1101)		Meta-analysis (3013)		
Model II	Mean difference (95% CI)	P-value	Mean difference (95% CI)	P-value	Mean difference (95% CI)	P-value	P-_Het_
Betaine	0.140 (-0.008 ; 0.288)	0.064	-0.046 (-0.202 ; 0.111)	0.568	0.049 (-0.133 ; 0.231)	0.598	0.090
Carnitine	0.074 (-0.122 ; 0.269)	0.460	-0.003 (-0.206 ; 0.199)	0.975	0.037 (-0.104 ; 0.177)	0.610	0.592
Choline	-0.112 (-0.289 ; 0.066)	0.218	**-0.258(-0.448 ; -0.067)**	0.008*	**-0.181 (-0.323 ; -0.038)**	0.013*	0.270
Deoxycarnitine	0.054 (-0.105 ; 0.212)	0.507	-0.040 (-0.266 ; 0.187)	0.730	0.023 (-0.107 ; 0.153)	0.730	0.504
TMAO	0.009 (-0.049 ; 0.067)	0.752	-0.048 (-0.108 ; 0.011)	0.111	-0.020 (-0.075 ; 0.036)	0.494	0.179

Plasma levels of TMAO and its precursors (in µmol/l) were natural log-transformed. The G-factor reflects the first component of the principal component analysis including the delayed recall score of the Stroop interference test, the letter-digit-substitution test, the verbal fluency task, the Purdue pegboard test and the 15-word learning test. Associations are presented as adjusted mean differences (with 95% confidence interval (CI)). Model I is adjusted for age, sex, education, lipid lowering medication use, body mass index. Model II is additionally adjusted for total cholesterol, HDL-cholesterol, smoking, hypertension and history of coronary heart disease. Associations passing the nominal significance threshold (*p* < 0.05) are marked bold, associations passing the FDR threshold are denoted with a *. In order to pool estimates, we performed a random-effects meta-analysis using the inverse variance method and the DerSimonian-Laird estimator.

*Abbreviations* RS I-4 = Rotterdam Study cohort I, fourth visit; RS III-2 = Rotterdam Study cohort III, second visit; P-_Het_ = P-value for heterogeneity, CI = confidence interval, P-value = nominal p-value, FDR = false discovery rate

### Associations between plasma levels of TMAO, its precursors and brain MRI markers

**Fig. 1:**
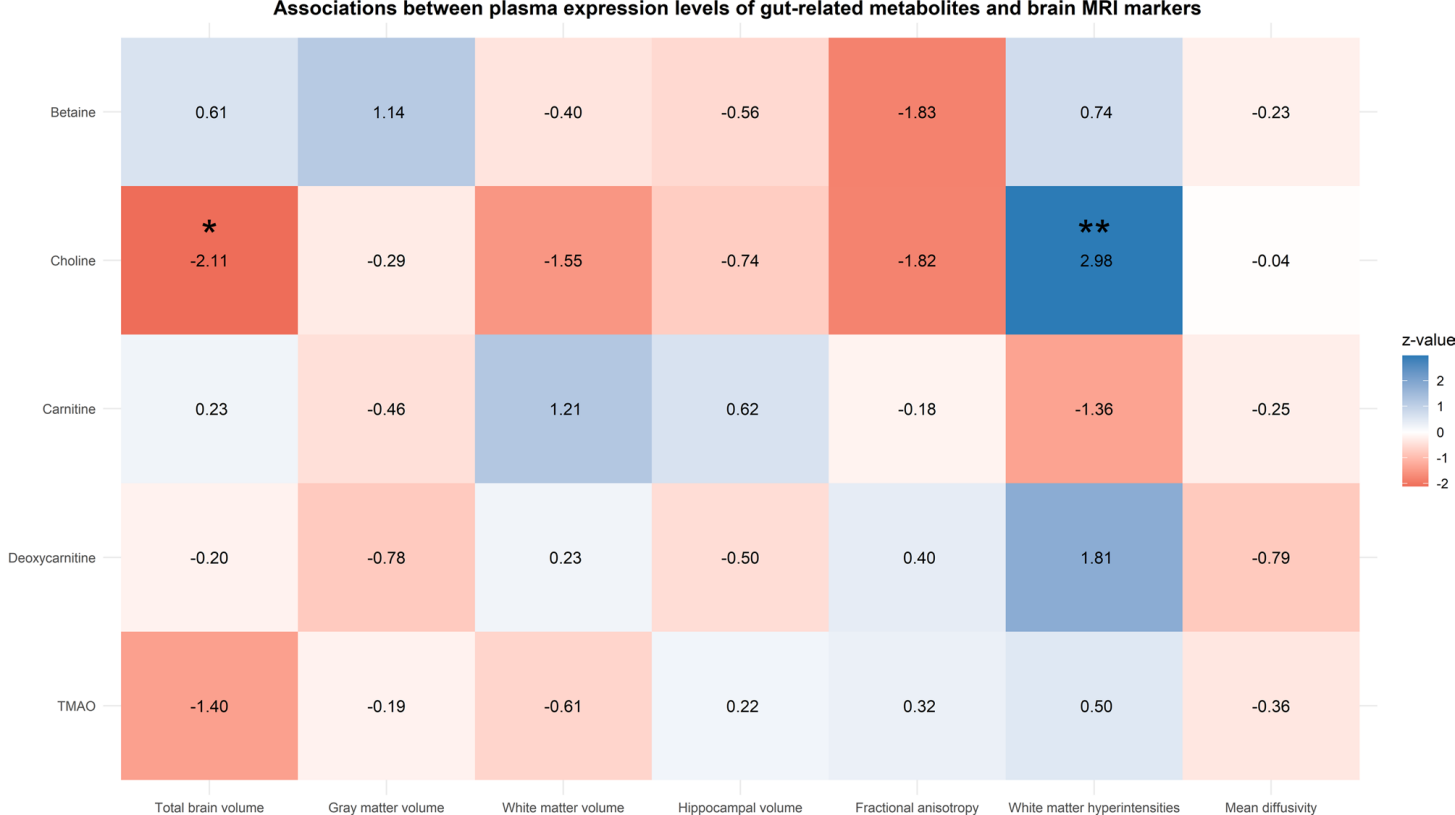
Pooled associations between plasma levels of TMAO, its precursors and neuroimaging markers are displayed as z-values (β/SE) and derived from the meta-analysis of linear regression models (A-B). Metabolites were log transformed for normalization purposes and all neuroimaging markers were standardized to facilitate comparison. Presented data refer to model 1, which is adjusted for age, sex, study cohort, education, intracranial volume, normal appearing white matter volume, time difference, lipid lowering medication use and body mass index. Stars denote significant associations: * = *p* < 0.05, ** = *p* < 0.01, with the latter one passing the false discovery rate (FDR) threshold. Extended details are provided in Table S1 of the Supplementary.

Although no associations were found between plasma levels of TMAO and brain MRI markers, several associations were observed with its precursors in the pooled associations of the RS and LLS. Higher plasma choline concurred with a smaller total brain volume (model I: -0.072 [95% CI: -0.140;-0.004], *P* = 0.037, P-_Het_=0.604), primarily driven by white matter, details of which are displayed in Fig. 1 and Table S1-A. Besides their association with brain atrophy, higher choline levels were also significantly associated with a higher white matter hyperintensity volume (model I: 0.237 [95% CI: 0.076;0.397], *P* = 0.004, P-_Het_=0.540, Fig. 1 and Table S1-A), even after adjustment for potential confounders and multiple testing correction (Table S1-B). In contrast, higher levels of carnitine were associated with lower white matter hyperintensity volume (model I: -0.177 [95% CI: -0.343;-0.010], *P* = 0.037, P-_Het_=0.386, Table S1-A). No associations were found with gray or white matter volume, hippocampal volumes, fractional anisotropy or mean diffusivity during meta-analysis.

#### Associations between plasma levels of TMAO, its precursors and incident dementia

Over a median follow-up of 11.3 years [interquartile range (IQR): 6.2–13.8], 500 participants developed incident dementia, of whom 391 had Alzheimer’s disease. No significant associations were found between TMAO, its precursors and the risk of incident dementia (Table 3).

**Table 3:** Associations between plasma levels of TMAO, its precursors and incident dementia

	Incident dementia			Incident Alzheimer’s disease		
Model I	*n*/*N*	HR (95% CI)	*P*-value	*n*/*N*	HR (95% CI)	*P*-value
Betaine	500/2451	0.93 (0.65 ; 1.31)	0.66	391/2451	0.92 (0.62 ; 1.36)	0.67
Carnitine	500/2451	0.87 (0.55 ; 1.36)	0.54	391/2451	0.86 (0.52 ; 1.42)	0.55
Choline	500/2451	0.96 (0.63 ; 1.46)	0.86	391/2451	0.80 (0.50 ; 1.29)	0.36
Deoxycarnitine	500/2451	1.13 (0.76 ; 1.68)	0.55	391/2451	1.04 (0.67 ; 1.62)	0.86
TMAO	500/2451	0.97 (0.85 ; 1.12)	0.71	391/2451	0.95 (0.82 ; 1.12)	0.56
Model II	n/N	HR (95% CI)	P-value	n/N	HR (95% CI)	P-value
Betaine	483/2359	0.94 (0.66 ; 1.34)	0.72	381/2359	0.92 (0.61 ; 1.37)	0.67
Carnitine	483/2359	0.84 (0.53 ; 1.34)	0.46	381/2359	0.84 (0.49 ; 1.41)	0.50
Choline	483/2359	0.94 (0.61 ; 1.45)	0.79	381/2359	0.77 (0.48 ; 1.25)	0.30
Deoxycarnitine	483/2359	1.12 (0.75 ; 1.68)	0.58	381/2359	1.03 (0.66 ; 1.60)	0.91
TMAO	483/2359	0.98 (0.85 ; 1.13)	0.78	381/2359	0.96 (0.82 ; 1.12)	0.58

*Note* Models pertaining to incident dementia only include participants from RS I-4 = Rotterdam Study cohort I, fourth visit. Plasma levels of TMAO and its precursors (in µmol/l) were natural log-transformed. Associations with incident dementia are presented as hazard ratios (HR), with 95% confidence interval (CI). Model I is adjusted for age, sex, education, lipid lowering medication use and body mass index. Model II is additionally adjusted for total cholesterol, HDL-cholesterol, smoking, hypertension and history of coronary heart disease. Associations passing the nominal significance threshold (*p* < 0.05) are marked bold, associations passing the FDR threshold are denoted with a *.

*Abbreviations* CI = confidence interval, P-value = nominal p-value, FDR = false discovery rate, n/N = number of incident cases/number of included participants, HR = hazard ratio

### Stratified, sensitivity and supplementary analyses

**Fig. 2:**
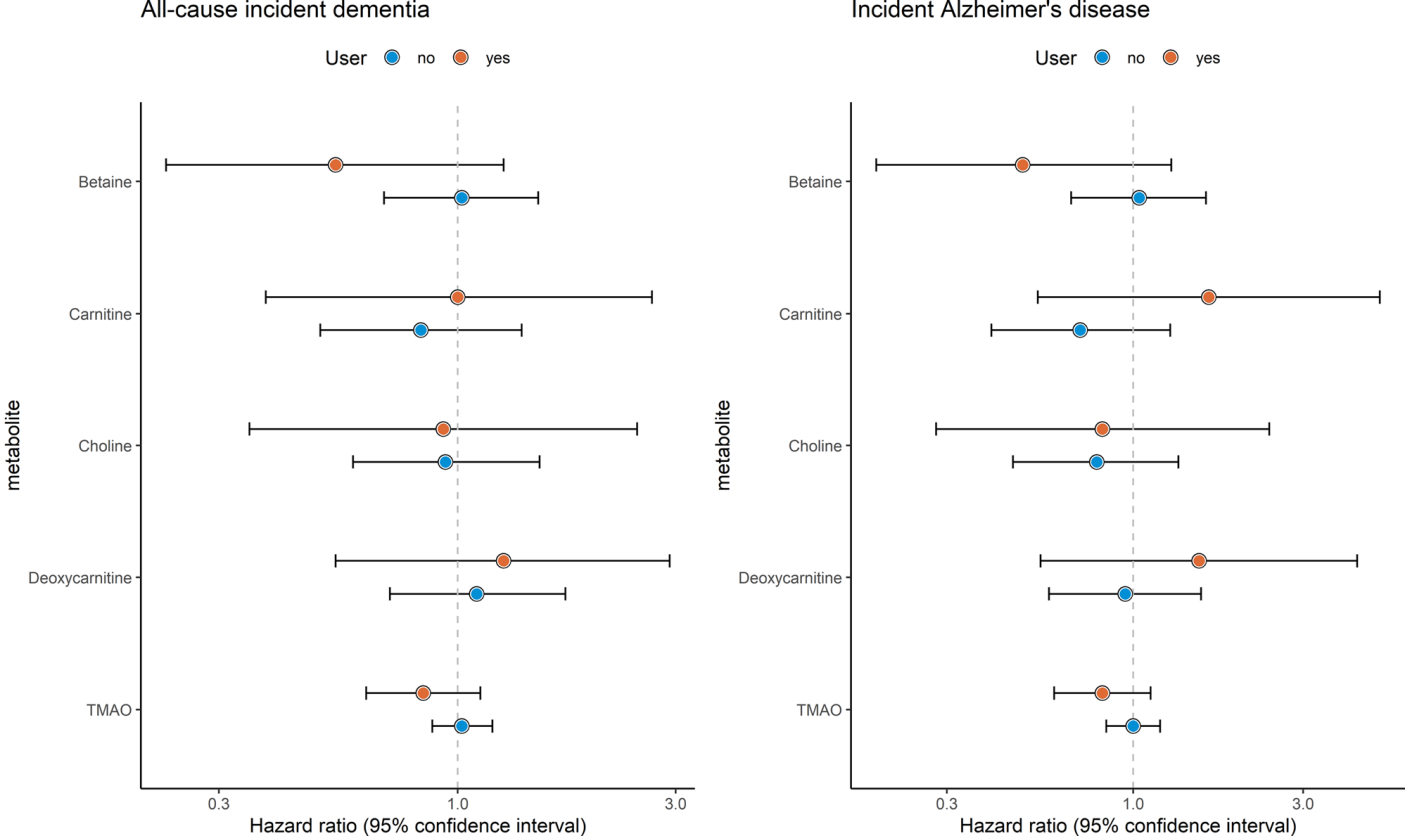
Associations between TMAO, its precursors and incident dementia, as well as Alzheimer’s disease, stratified by non-users (user = no) and users (user = yes) of antibiotics or proton pump inhibitors. Among 2,024 non-users of antibiotics or proton pump inhibitors, 413 participants developed incident (all-cause) dementia, from which 321 individuals had Alzheimer’s disease. Among 427 users of antibiotics or proton pump inhibitors, 87 participants developed incident (all-cause) dementia, from which 70 individuals had Alzheimer’s disease. Effect estimates from Cox proportional hazard models are presented as hazard ratios (HR) with 95% confidence intervals

Stratification by participants who used antibiotics or PPIs (*n* = 427) and those who did not (*n* = 2,024), yielded no notable differences in associations of plasma TMAO and its precursors with incident dementia (Fig. 2). Only among the small subset of participants with impaired renal function (eGFR < 60 mL/min, *n* = 245), TMAO was significantly associated with an increased risk of incident dementia (model I HR: 1.73 [95% CI: 1.16;2.60, *P* = 0.01], including Alzheimer’s disease (Table S2). Associations did not differ by sex (Figure S3). Precursors of TMAO were moderately correlated (Pearson’s correlation coefficient (PCC) for betaine and choline 0.36, Figure S4), followed by carnitine and deoxycarnitine (PCC: 0.35) and choline and deoxycarnitine (PCC: 0.31). Scatterplots of plasma TMAO and its precursors and neuroimaging markers are provided in Figure S5.

## Discussion

In this population-based study, we could not confirm an association between plasma levels of TMAO and cognition, neuroimaging markers or incident dementia, except in those with impaired renal function. However, we did find that choline, the main precursor of TMAO, was associated with lower cognition, lower total brain volume and a higher white matter hyperintensity volume. In contrast, higher levels of carnitine concurred with a lower white matter hyperintensity volume. No other precursors of TMAO showed associations with cognition, neuroimaging markers or incident dementia.

Choline is a key component for the synthesis of acetylcholine; a neurotransmitter recognized for its role in memory and learning. While dietary choline has predominantly been attributed positive effects on cognition in early life [36], recent research suggests that depending on the composition and diversity of the gut microbiota, other pathways of choline metabolism may be activated as well, resulting in the synthesis of betaine, sphingomyelin and phosphatidylcholine (components of the cell membrane structure) or TMAO [37]. With dysbiosis of the gut, the probability of choline being metabolized into TMAO increases [36], which has previously been associated with both cardiovascular outcomes [6, 8] and cognitive impairment [38]. Several animal and human studies indicate that TMAO has deleterious effects on synaptic plasticity [39] and overall cognitive performance [9], but most of them did not consider the bioavailability of its main precursor, choline. A multi-ethnic, population-based cohort study where both TMAO and its precursors were studied in relation to inflammatory and cardiometabolic risk biomarkers, showed that choline, rather than TMAO, was associated with an adverse cardiovascular risk profile [40]. This was also observed in other population-based cohorts that examined such associations separately: while some found no association between TMAO and atherosclerosis [41, 42], others reported an increased risk of cardiometabolic disease and mortality with higher levels of choline [43, 44]. Given that cardiovascular disease and cognitive impairment share common pathways, findings of this study add to the hypothesis that choline, rather than TMAO, may be one of the underlying pathological substrates.

Evidence on the relationship between plasma TMAO, its precursors and structural brain imaging markers of dementia is limited and conflicting. It also remains uncertain whether TMAO, or its precursor trimethylamine (TMA), alters the integrity of the blood-brain-barrier [45]. In a cross-sectional analysis of a multicenter hospital-based cohort study among stroke patients, both TMAO and choline were associated with a higher white matter hyperintensity volume [12], which we could only confirm for choline in our study. By contrast, in the Framingham Offspring cohort, a higher (self-reported) intake of choline was associated with little to no white matter hyperintensities [46]. It is postulated that the association of choline with cerebrovascular disease may be dependent on the morphology (i.e. small vs. large vessel disease), as well as the cardiovascular risk profile of the population [47]. Within our study, higher choline levels showed a pattern with lower white matter volume, diminished fractional anisotropy, and a higher white matter hyperintensity volume, which suggests a connection to small vessel disease. It is also worth noting that our study included an older population of adults with relatively more cardiovascular comorbidity (RS-I-4 and RS-III-2) and an external validation cohort of younger individuals with less cardiovascular comorbidity (LLS), and we indeed observed some heterogeneity on the association of TMAO and its precursors with brain volumes and white matter hyperintensity volume. Perhaps this points towards an age-dependent effect of TMAO and its precursors on the brain, due to a change in composition or diversity of gut microbiota in aging individuals. It could also be that plasma levels of these metabolites differ from those in cerebrospinal fluid [10], the latter being a more direct reflection of biochemical processes occurring within the central nervous system, whereas plasma metabolites can be influenced by various factors associated with gut microbiota. The protective association between carnitine and white matter hyperintensity volume that we found has only been reported in haemodialysis patients [48] and some animal studies [49, 50], supporting the notion that carnitine may also have an important role in the gut-brain axis.

Longitudinal associations of TMAO and its precursors with dementia have recently been described among older adults aged 65 years and older in the Cardiovascular Health Study [51]. In line with our findings, they found no associations between TMAO, carnitine, choline, or betaine and incident dementia, but identified associations with other TMAO-related metabolites including crotonobetaine and γ-butyrobetaine, both of which were not measured in this study. Our sensitivity analysis indicates that only in a small subset of participants with impaired clearance (eGFR < 60 mL/min), there was an increased risk of dementia with higher levels of TMAO, while no associations were found in the overall sample. Collectively, the results of our study suggest that in community dwelling individuals, who generally have retained renal function, the precursors of TMAO, rather than TMAO itself, may be involved in the pathophysiology of dementia.

### Strengths and limitations

Strengths of this study include a population-based study design, with a large sample of community-dwelling adults that underwent standardized assessment of TMAO and its precursors, an extensive neuropsychological test battery, good quality brain MRI and more than a decade of follow-up for incident dementia. In addition, we included an external validation cohort (LLS) with younger individuals for neuroimaging associations. Yet, our study had several limitations that should be mentioned. First, due to the cross-sectional nature of associations between TMAO, its precursors, cognition and brain MRI markers, we cannot infer the temporality of associations. Temporal changes in the composition and diversity of gut-microbiota may have a considerable effect on the association of TMAO and its precursors with cognition and brain structure, highlighting the need for longitudinal studies with repeated assessments. Second, information on diet and composition of gut-microbiota was not available in this study, which could be a source of residual confounding. In addition, use of hormone replacement therapy (HRT) in post-menopausal women is thought to alter gut-microbiota and related metabolites, although evidence for this is limited [52]. We did not have information on HRT use at our disposal, therefore we cannot ascertain to what extent this may have influenced our results. Third, since renal function data was not available for the whole cohort (only for 33% of participants), we cannot infer to which extent this may have had consequences for the excretion of TMAO or its precursors. Fourth, although the LLS served as an independent validation cohort for neuroimaging associations in the RS, we cannot rule out the possibility that this cohort is enriched with genes for longevity, which could have affected our results. Finally, the findings from our predominantly White population may not generalize to other ethnicities.

## Conclusion

In this population-based study, we found that higher levels of plasma choline, rather than TMAO, were associated with cognitive impairment, brain atrophy and markers of cerebral small vessel disease. TMAO and its precursors showed no associations with incident dementia, except in individuals with impaired kidney function. Our findings exemplify that relationships between TMAO and its precursors are intricate, and that these should be studied in tandem to elucidate mechanisms along the gut-brain axis.

### Electronic supplementary material

Below is the link to the electronic supplementary material.

Supplementary Material 1

## Data Availability

Reasonable requests for data sharing can be directed towards the Rotterdam Study Management Team (secretariat.epi@erasmusmc.nl), who follow a protocol to approve data requests. Due to privacy regulations and informed consent of participants, data cannot be uploaded on a public repository.

## Declarations

### Summary for social media if published

@ErasmusMC:  In the population-based Rotterdam Study, associations were explored between plasma TMAO & its precursors with cognition, neuroimaging markers and dementia. Notably, higher levels of plasma choline were linked to cognitive impairment, brain atrophy and markers of cerebral small vessel disease. This research underscores the complex dynamics of the gut-brain axis and the crucial need for further studies in this field. #GutMicrobiome #Neuroimaging #DementiaResearch.
