# Supplementary material for: Plasma trimethylamine N-oxide (TMAO): associations with cognition, neuroimaging, and dementia
Source: Alzheimers Res Ther. 2024 May 20;16:113. doi: 10.1186/s13195-024-01480-1 (PMC11103865; doi:10.1186/s13195-024-01480-1)
Supplement: Supplementary file 1 — Supplementary Material 1 [file 13195_2024_1480_MOESM1_ESM.docx]

**Supplementary file – Plasma Trimethylamine N-oxide (TMAO): Associations with Cognition, Neuroimaging, and Dementia**

*Supplementary text, tables and figures*

| *Content* | *Page number* |
| --- | --- |
| Methods S1: Brief description of the Rotterdam Study and the Leiden Longevity Study | 2 |
| Methods S2: Analytical techniques used for plasma TMAO and its precursors | 3 |
| Methods S3: Neuroimaging protocols in the RS and LLS | 4 |
| Methods S4: Assessment of covariates | 7 |
| Figure S1: Flowchart of study design | 8 |
| Figure S2: Relative plasma levels of TMAO and its precursors per G-factor category | 9 |
| Table S1: Associations between plasma levels of TMAO, its precursors and brain MRI markers (model I (S1-A) and model II (S1-B)) | 10 |
| Table S2: Associations between plasma levels of TMAO, its precursors and incident dementia with impaired renal function | 14 |
| Figure S3: Associations between TMAO, its precursors and incident dementia, as well as Alzheimer’s disease, stratified by sex | 15 |
| Figure S4: Correlation between plasma levels of TMAO and its precursors | 16 |
| Figure S5: Scatterplots of plasma TMAO and its precursors and neuroimaging markers | 17 |

**Methods S1: Brief description of the Rotterdam Study and the Leiden Longevity Study**

*Rotterdam Study*
The Rotterdam Study (RS) is a large, prospective, population-based cohort of the Netherlands including community-dwelling adults from Ommoord (a suburb of Rotterdam) that aims to unravel mechanisms and potential targets of intervention for chronic diseases in mid-life and late-life.^14^

In summary, all inhabitants of Ommoord aged ≥55 years were invited to participate in 1990, from which 7983 individuals enrolled (RS-I). Subsequently, the cohort was expanded thrice: first in 2000, which resulted in the inclusion of 3,011 individuals who had reached the eligible age or had moved into the study area (RS-II), later in 2006, with 3,932 individuals aged 45 or over (RS-III) and recently in 2015 with 3,005 individuals aged 40 or over (RS-IV). Participants take part in comprehensive interviews and visit the research facility for an in-person examination every 3-6 years. In addition, participants are monitored continuously though electronic linkage of medical records with the study database.

*Leiden Longevity Study*
The Leiden Longevity Study (LLS) is a family-based longitudinal cohort based in the Netherlands, including 421 families of long-lived European siblings, their offspring and partners. An extended description of the study design has been published previously.^15^ In summary, families were invited to participate if both siblings were alive and fulfilled the inclusion criteria, which were: being at least 89 years or older for men, or 91 years or older for women. No selection criteria for health or other demographic factors were applied. A total of 944 siblings, 1671 offspring and 744 spouses of the siblings were enrolled between 2002 and 2006. Participants of the LLS had assessments at the research center and were subsequently followed up.

**Methods S2: Analytical techniques used for plasma TMAO and its precursors**

Plasma samples were obtained from fasting participants of the RS and non-fasting participants of the LLS using ethylenediaminetetetacetic acid (EDTA) tubes. Each sample of 10 μL was spiked with an internal standard solution, after which samples were cooled to 10°C. Vials of 1.0 μL were then injected into the Liquid Chromatography (LC) tandem mass spectrometry system (Agilent 1290 Infinity II LC System) via a Accq-Tag Ultra column (Waters), at a flow of 0.7 mL/min over a 3 min gradient. TMAO and its precursors were quantified in a similar manner in the RS and LLS, using the triple quadrupole mass spectrometer (AB SCIEX Qtrap 650, version 3.0.2) to quantify Trimethylamine-N-oxide (TMAO), as well as its precursors betaine, carnitine, choline, and deoxycarnitine with the MultiQuant Software for Quantitative Analysis (AB SCIEX, Version 3.0.2).^16^ Analytes were observed in the positive ion mode in Multiple Reaction Monitoring (MRM) with nominal mass resolution. Internal standards were used to assign MRM peaks and achieve normalization. Batch effects were analyzed by principal component analysis of the log2 transformed ratios of compound intensity to internal standard intensity. All analyses were conducted at the Biomedical Metabolomics Facility in Leiden, the Netherlands.

**Methods S3: Neuroimaging protocols in the RS and LLS**
*MRI scan protocol Rotterdam Study (RS)*
A multi-sequence magnetic resonance imaging (MRI) protocol was introduced in the Rotterdam Study from 2005 and onwards, which included a single 1.5 Tesla scanner (General Electric Healthcare, Milwaukee, WI).^18^ This scanner included a 8-channel head coil and a high-resolution axial T1-weighted sequence, a T2*-weighted gradient echo sequence, a fluid-attenuated inversion recovery (FLAIR) sequence and a proton density (PD)–weighted sequence. A k-nearest neighbor tissue classification algorithm was used to estimate total brain volume (TBV), gray matter (GMV), white matter (WMV) and white matter hyperintensities (WMH),^19^ which were then inspected by trained researchers and manually corrected if needed. Hippocampal volumes (HV) were analysed by FreeSurfer 6.1.18. This MRI protocol was expanded in 2006 and onwards with a diffusion-weighted imaging (DWI) sequence,^20^ using eddy current, head motion correction and fitting of diffusion tensors as pre-processing steps. An extensive description of MRI acquisition, segmentation methods and related metrics, preprocessing steps, assessment of voxelwise white matter integrity and global white matter integrity is available elsewhere.^21^ In brief, DWI was conducted using a single-shot, spin echo echo-planar imaging sequence with the following parameters: TR = 8575 ms, TE = 82.6 ms, axial FOV = 210 × 210 mm, matrix = 96 × 64 (phase encoding) (zero-padded to 256 × 256) slice thickness = 3.5 mm, and 35 contiguous slices. The maximum b-value was 1000 s/mm² in 25 noncollinear directions, with three volumes acquired without diffusion weighting (b-value = 0 s/mm²).^20^ The diffusion data were pre-processed according to a standardized pipeline,^21^ including correction for motion and eddy currents by affine coregistration, using the Elastix software.^22,23^ Images were resampled to a 1.0 mm isotropic resolution and non-brain tissue was removed using FSL's Brain Extraction Tool.^24^ Tensor fits were performed by a nonlinear Levenberg Marquardt estimator, through the ExploreDTI software.^25^ Data quality was assessed visually by examining axial FA slices at 4 mm intervals, along with two coronal and two sagittal slices around the brain's center. A validated k-nearest-neighbor brain tissue classification method was applied to categorize voxels into cerebrospinal fluid (CSF), gray matter, normal-appearing white matter (NAWM), and WMH, with volumes (measured in milliliters) calculated based on these classifications.^19,26^ The NAWM was considered as the white matter volume without WMH, details of which were described previously.^20,27^ Fractional anisotropy (FA) and mean diffusivity (MD) were only assessed in NAWM. Lower estimates of FA and higher estimates of MD suggest diminished integrity of white matter.

*MRI scan protocol Leiden Longevity Study (LLS)*
In the LLS, neuroimaging was performed on a whole-body magnetic resonance system with a field strength of 3-T (Philips Medical Systems). Three-dimensional images on T1-weighted, T-2 weighted, T2*-weighted and FLAIR sequences were used to determine TBV, GMV, WMV, HV and WMH. Settings for the imaging parameters have been published previously.^28^ The Medical Imaging Processing, Analysis and Visualization (MIPAV) software^29^ was deployed for all MRI scans, including a skull-stripping processing step using the BET (Brain Extraction Tool). The libraries used to quantify brain volumes included the FSL-Tool Structural Image Evaluation (for TBV, GMV and WMV) with Normalization of Atrophy (SIENAX) and the FMRIB's Registration and Segmentation Tool for hippocampal volumes. Automated segmentation of WMH volume was conducted using Software for Neuro-Image Processing in Experimental Research, an in-house program developed for image processing.^30^ This segmentation relied on information from T2-weighted and FLAIR images. WMH were identified as hyperintense lesions on both proton density and T2-weighted images. Lesions connected to the lateral ventricles were classified as periventricular WMH, while those not connected to the lateral ventricles were classified as deep WMH. According to previously described methods,^31^ WMHs were identified by two independent reviewers, who were blinded to the characteristics of study participants. The raw diffusion tensor images were pre-processed using FDT (FMRIB's Diffusion Toolbox) tools and corrected for effects of head movement and eddy currents in the gradient coils, using the non–diffusion-weighted images for reference volume. The imaging parameters for DTI images were: TR = 9,592 milliseconds, TE = 56 milliseconds, FA =90°, FOV = 220 x 220 x 128 mm, matrix size 112 x 110, 64 transverse slices to cover the entire brain with a slice thickness of 2 mm with no gap between slices, 32 measurement directions, and a b-value = 1,000. Images were aligned into common space by the linear registration tool FLIRT (linear registration tool of the FMRIB), with subsequent extraction of mean FA and MD values. A conservative Montreal Neurological Institute (MNI) white matter mask was used to extract the white matter from the FLAIR image. Following the exclusion of the cerebellum and brainstem, a threshold was established to identify hyper-intense white matter voxels.

For both RS and LLS, NAWM and WMHs were assessed using automated segmentation methods.^26,30^ Briefly, every voxel was automatically classified into a specific brain tissue category, and the voxel count was then totaled and multiplied by the voxel size to determine the volumes of NAWM and WMH. FA and MD values were assessed within NAWM excluding regions affected by WMH. This approach allows for a more specific evaluation of microstructural changes in white matter before the onset of macrostructural damage represented by WMH.

**Methods S4: Assessment of covariates**
Assessment of covariates was comparable between the RS and LLS. Information on educational attainment and smoking status was obtained from standardized interviews. Educational attainment was categorized as primary, further and higher education. Participants were categorized into never, former or current smokers, based on current habits. Medical history was retrieved from medical records (LLS) and/or continuous linkage of health data from general practitioners to the study database (RS). Data on medication that may influence the gut microbiome (current use of antibiotics, lipid lowering medication, e.g.) was collected during home-interviews in the RS, whereas this information was obtained from pharmacy data in the LLS. Subsequently, participants were categorized into non-users and users. A history of CHD was characterized as a history of myocardial infarction, percutaneous coronary intervention or coronary artery bypass grafting.

Blood pressure was measured during visits at the research center for all participants. Hypertension was defined as a pressure of ≥140/90 mmHg and/or the use of blood pressure lowering drugs. The body mass index (BMI) was calculated by dividing weight in kilograms by height in meters squared. Blood samples that were taken during center visits also provided information on serum total cholesterol, high-density lipoprotein cholesterol and glucose levels. Hypercholesterolemia was classified as a total cholesterol value of ≥6.2 mmol/L in serum (using the automated assays Boehringer Mannheim System in RS, and the Hitachi 747 and 911 in LLS) and/or use of lipid-lowering medication. The presence of type II diabetes was based on serum glucose levels using the same assays and was determined with a fasting glucose level ≥7.0 mmol/L (126 mg/dL), a nonfasting serum glucose level ≥11.1 mmol/L (200 mg/dL), and/or the use of blood glucose-lowering medication.

**Figure S1: Flowchart of the study design**

**
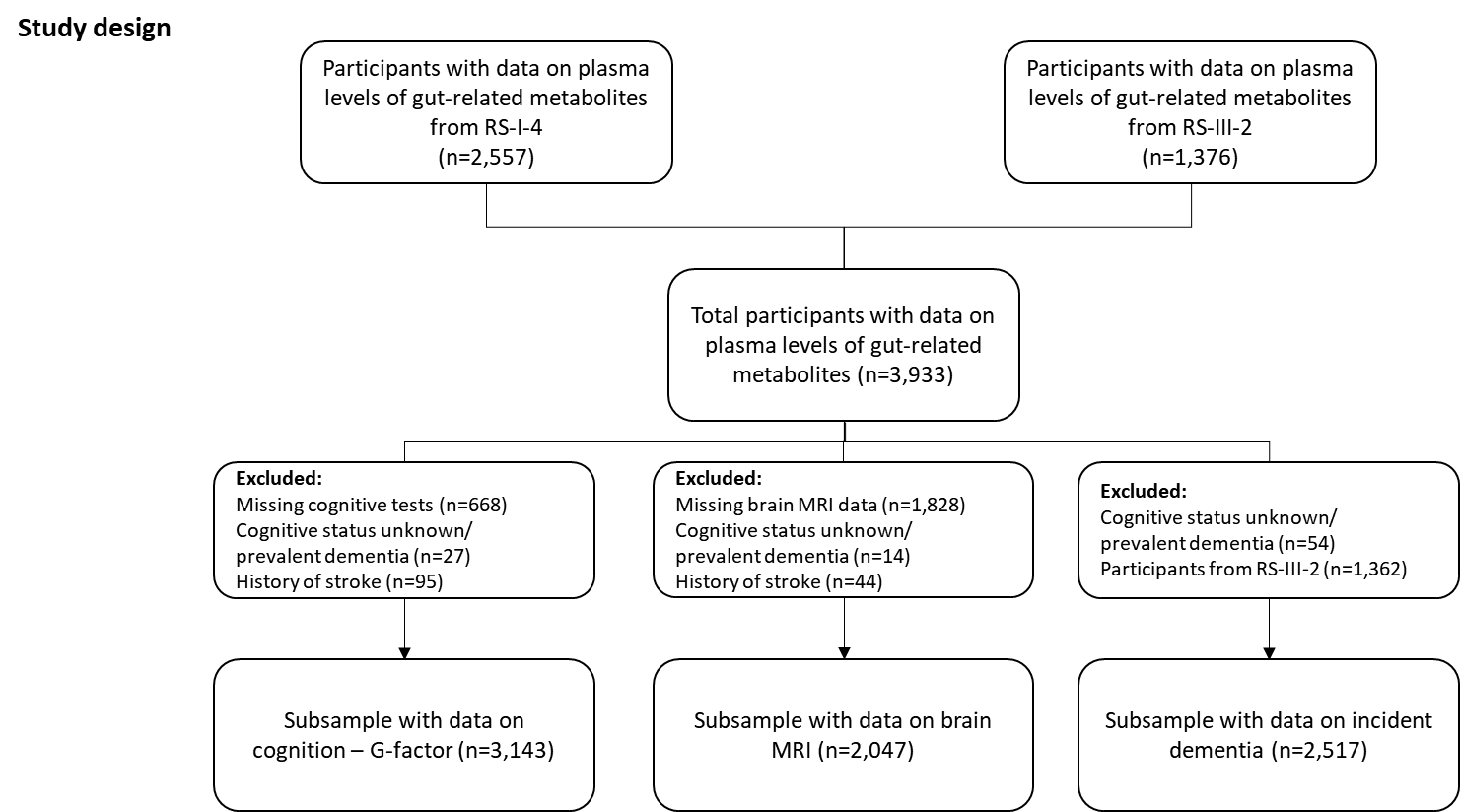
**

**Figure S1: Flowchart study design:** RS I-4 = Rotterdam Study cohort I, fourth visit; RS III-2 = Rotterdam Study cohort III, second visit. Note that the eventual amount of participants included in each model depends on the completeness of covariates. For replication purposes, the neuroimaging sample of the Rotterdam Study was extended with 318 participants from the Leiden Longevity Study, who also had the required data available.

**Figure S2: Relative plasma levels of TMAO and its precursors per G-factor category**

**Figure S2:** This cluster plot displays the mean relative plasma levels of TMAO and its precursors per G-factor category. The x-axis denotes the G-factor categories from severe cognitive dysfunction (far left, <-2SD) to cognitive function above average (far right, >2SD). Low levels of these gut-related metabolites are displayed in blue, high levels are displayed in red.

**Table S1-A. Associations between plasma levels of TMAO, its precursors and brain MRI markers (model I)**

| Total brain volume (per SD) | | | | | | | | | |
| --- | --- | --- | --- | --- | --- | --- | --- | --- | --- |
|  | RS I-4 (N=932) | | RS III-2 (N=1097) | | LLS  (N=282) | | Meta-analysis (N=2311) | | |
| Model I | Mean difference (95% CI) | P-value | Mean difference (95% CI) | P-value | Mean difference (95% CI) | P-value | Mean difference (95% CI) | P-value | P-_Het_ |
| Betaine | 0.007 (-0.076 - 0.091) | 0.866 | 0.025 (-0.047 - 0.097) | 0.496 | -0.368 (-1.095 - 0.359) | 0.330 | 0.015 (-0.040 - 0.070) | 0.588 | 0.556 |
| Carnitine | -0.053 (-0.163 - 0.058) | 0.351 | 0.076 (-0.017 - 0.169) | 0.109 | -0.227 (-1.006 - 0.553) | 0.574 | 0.011 (-0.100 - 0.122) | 0.843 | 0.173 |
| Choline | **-0.109 (-0.212 - -0.005)** | **0.040** | -0.047 (-0.137 - 0.043) | 0.308 | 0.126 (-0.692 - 0.943) | 0.765 | **-0.072 (-0.140 - -0.004)** | **0.037** | 0.604 |
| Deoxycarnitine | -0.070 (-0.153 - 0.013) | 0.098 | 0.053 (-0.048 - 0.153) | 0.305 | 0.005 (-0.943 - 0.953) | 0.992 | -0.013 (-0.114 - 0.087) | 0.798 | 0.177 |
| TMAO | -0.023 (-0.056 - 0.009) | 0.160 | -0.008 (-0.034 - 0.018) | 0.558 | 0.235 (-0.012 - 0.481) | 0.073 | -0.009 (-0.047 - 0.028) | 0.629 | 0.113 |
| Gray matter volume (per SD) | | | | | | | | | |
|  | RS I-4 (N=932) | | RS III-2 (N=1097) | | LLS  (N=282) | | Meta-analysis (N=2311) | | |
| Model I | Mean difference (95% CI) | P-value | Mean difference (95% CI) | P-value | Mean difference (95% CI) | P-value | Mean difference (95% CI) | P-value | P-_Het_ |
| Betaine | 0.025 (-0.126 - 0.175) | 0.748 | 0.076 (-0.047 - 0.198) | 0.227 | -0.892 (-2.249 - 0.464) | 0.208 | 0.050 (-0.052 - 0.151) | 0.338 | 0.345 |
| Carnitine | **-0.229 (-0.427 - -0.030)** | **0.024** | 0.078 (-0.081 - 0.237) | 0.336 | -0.585 (-2.132 - 0.962) | 0.465 | -0.085 (-0.364 - 0.195) | 0.552 | 0.048 |
| Choline | -0.130 (-0.317 - 0.056) | 0.171 | 0.060 (-0.095 - 0.215) | 0.446 | -0.138 (-1.750 - 1.474) | 0.868 | -0.023 (-0.164 - 0.117) | 0.745 | 0.303 |
| Deoxycarnitine | -0.100 (-0.249 - 0.050) | 0.191 | 0.022 (-0.150 - 0.195) | 0.799 | 0.561 (-1.303 - 2.425) | 0.560 | -0.046 (-0.158 - 0.067) | 0.426 | 0.470 |
| TMAO | -0.058 (-0.117 - 0.001) | 0.052 | 0.036 (-0.008 - 0.081) | 0.109 | 0.390 (-0.110 - 0.889) | 0.138 | 0.005 (-0.094 - 0.104) | 0.917 | 0.014 |
| White matter volume (per SD) | | | | | | | | | |
|  | RS I-4 (N=932) | | RS III-2 (N=1097) | | LLS  (N=282) | | Meta-analysis (N=2311) | | |
| Model I | Mean difference (95% CI) | P-value | Mean difference (95% CI) | P-value | Mean difference (95% CI) | P-value | Mean difference (95% CI) | P-value | P-_Het_ |
| Betaine | -0.011 (-0.170 - 0.148) | 0.892 | -0.029 (-0.165 - 0.106) | 0.673 | -0.886 (-2.157 - 0.384) | 0.183 | -0.027 (-0.130 - 0.076) | 0.605 | 0.407 |
| Carnitine | 0.125 (-0.085 - 0.336) | 0.243 | 0.053 (-0.122 - 0.229) | 0.553 | 0.058 (-1.411 - 1.526) | 0.939 | 0.082 (-0.051 - 0.216) | 0.227 | 0.874 |
| Choline | -0.059 (-0.257 - 0.139) | 0.560 | -0.134 (-0.304 - 0.037) | 0.125 | 0.203 (-1.311 - 1.716) | 0.795 | -0.100 (-0.229 - 0.029) | 0.128 | 0.790 |
| Deoxycarnitine | -0.023 (-0.182 - 0.135) | 0.774 | 0.066 (-0.124 - 0.256) | 0.494 | -0.006 (-1.768 - 1.757) | 0.995 | 0.013 (-0.108 - 0.135) | 0.828 | 0.780 |
| TMAO | 0.015 (-0.047 - 0.078) | 0.629 | -0.047 (-0.096 - 0.002) | 0.063 | 0.364 (-0.106 - 0.834) | 0.141 | -0.008 (-0.085 - 0.069) | 0.837 | 0.085 |
| Hippocampal volume (per SD) | | | | | | | | | |
|  | RS I-4 (N=869) | | RS III-2 (N=986) | | LLS  (N=282) | | Meta-analysis (N=2137) | | |
| Model I | Mean difference (95% CI) | P-value | Mean difference (95% CI) | P-value | Mean difference (95% CI) | P-value | Mean difference (95% CI) | P-value | P-_Het_ |
| Betaine | -0.009 (-0.197 - 0.179) | 0.928 | -0.052 (-0.208 - 0.105) | 0.518 | -1.357 (-2.721 - 0.007) | 0.062 | -0.059 (-0.256 - 0.138) | 0.558 | 0.157 |
| Carnitine | 0.045 (-0.204 - 0.294) | 0.723 | 0.054 (-0.146 - 0.254) | 0.597 | 1.257 (-0.301 - 2.815) | 0.125 | 0.066 (-0.111 - 0.242) | 0.466 | 0.319 |
| Choline | -0.030 (-0.266 - 0.206) | 0.803 | -0.073 (-0.267 - 0.122) | 0.464 | 0.174 (-1.506 - 1.854) | 0.841 | -0.054 (-0.203 - 0.095) | 0.480 | 0.929 |
| Deoxycarnitine | -0.057 (-0.241 - 0.128) | 0.547 | -0.006 (-0.223 - 0.211) | 0.958 | **1.976 ( 0.169 - 3.784)** | **0.041** | 0.012 (-0.262 - 0.287) | 0.931 | 0.088 |
| TMAO | 0.015 (-0.059 - 0.089) | 0.697 | 0.000 (-0.055 - 0.055) | 0.991 | 0.323 (-0.206 - 0.852) | 0.242 | 0.007 (-0.037 - 0.052) | 0.739 | 0.478 |
| White matter hyperintensities (per SD) | | | | | | | | | |
|  | RS I-4 (N=932) | | RS III-2 (N=1097) | | LLS  (N=259) | | Meta-analysis (N=2288) | | |
| Model I | Mean difference (95% CI) | P-value | Mean difference (95% CI) | P-value | Mean difference (95% CI) | P-value | Mean difference (95% CI) | P-value | P-_Het_ |
| Betaine | 0.012 (-0.199 - 0.224) | 0.909 | 0.068 (-0.093 - 0.230) | 0.406 | 0.151 (-1.620 - 1.922) | 0.869 | 0.048 (-0.080 - 0.176) | 0.461 | 0.912 |
| Carnitine | -0.021 (-0.302 - 0.260) | 0.884 | **-0.259 (-0.468 - -0.051)** | **0.015*** | -0.581 (-2.901 - 1.739) | 0.628 | **-0.177 (-0.343 - -0.010)** | **0.037** | 0.386 |
| Choline | 0.205 (-0.058 - 0.468) | 0.126 | **0.267 ( 0.064 - 0.471)** | **0.010*** | -0.836 (-2.848 - 1.175) | 0.424 | **0.237 ( 0.076 - 0.397)** | **0.004*** | 0.540 |
| Deoxycarnitine | 0.127 (-0.084 - 0.338) | 0.237 | 0.157 (-0.069 - 0.384) | 0.174 | -0.191 (-2.527 - 2.145) | 0.874 | 0.139 (-0.014 - 0.293) | 0.074 | 0.945 |
| TMAO | -0.005 (-0.088 - 0.078) | 0.914 | 0.021 (-0.038 - 0.080) | 0.484 | 0.060 (-0.586 - 0.705) | 0.858 | 0.012 (-0.035 - 0.060) | 0.608 | 0.872 |
| Fractional anisotropy (per SD) | | | | | | | | | |
|  | RS I-4 (N=755) | | RS III-2 (N=1071) | | LLS  (N=259) | | Meta-analysis (N=2085) | | |
| Model I | Mean difference (95% CI) | P-value | Mean difference (95% CI) | P-value | Mean difference (95% CI) | P-value | Mean difference (95% CI) | P-value | P-_Het_ |
| Betaine | -0.130 (-0.412 - 0.152) | 0.365 | -0.167 (-0.370 - 0.037) | 0.108 | 0.703 (-1.147 - 2.552) | 0.465 | -0.148 (-0.312 - 0.017) | 0.079 | 0.650 |
| Carnitine | -0.226 (-0.597 - 0.145) | 0.231 | 0.113 (-0.150 - 0.377) | 0.399 | -0.333 (-2.336 - 1.671) | 0.748 | -0.016 (-0.257 - 0.226) | 0.898 | 0.325 |
| Choline | -0.220 (-0.564 - 0.124) | 0.210 | -0.174 (-0.431 - 0.082) | 0.182 | -0.092 (-2.077 - 1.894) | 0.929 | -0.189 (-0.394 - 0.015) | 0.069 | 0.974 |
| Deoxycarnitine | 0.153 (-0.111 - 0.417) | 0.254 | -0.075 (-0.360 - 0.210) | 0.605 | -0.624 (-2.935 - 1.686) | 0.602 | 0.043 (-0.149 - 0.236) | 0.659 | 0.437 |
| TMAO | 0.086 (-0.023 - 0.196) | 0.121 | -0.035 (-0.110 - 0.039) | 0.349 | 0.205 (-0.437 - 0.847) | 0.538 | 0.021 (-0.082 - 0.124) | 0.684 | 0.167 |
| Mean diffusivity (per SD) | | | | | | | | | |
|  | RS I-4 (N=755) | | RS III-2 (N=1071) | | LLS  (N=259) | | Meta-analysis (N=2085) | | |
| Model I | Mean difference (95% CI) | P-value | Mean difference (95% CI) | P-value | Mean difference (95% CI) | P-value | Mean difference (95% CI) | P-value | P-_Het_ |
| Betaine | -0.035 (-0.270 - 0.201) | 0.773 | -0.006 (-0.160 - 0.149) | 0.944 | -0.576 (-2.129 - 0.977) | 0.476 | -0.019 (-0.148 - 0.110) | 0.777 | 0.764 |
| Carnitine | 0.147 (-0.163 - 0.457) | 0.351 | -0.188 (-0.388 - 0.011) | 0.064 | -1.240 (-3.021 - 0.541) | 0.187 | -0.082 (-0.427 - 0.263) | 0.642 | 0.092 |
| Choline | 0.003 (-0.285 - 0.290) | 0.986 | -0.006 (-0.200 - 0.189) | 0.955 | -0.872 (-2.590 - 0.845) | 0.331 | -0.011 (-0.171 - 0.149) | 0.895 | 0.613 |
| Deoxycarnitine | -0.161 (-0.382 - 0.059) | 0.151 | 0.018 (-0.198 - 0.235) | 0.867 | -1.018 (-2.945 - 0.908) | 0.313 | -0.077 (-0.247 - 0.092) | 0.370 | 0.329 |
| TMAO | -0.028 (-0.120 - 0.063) | 0.545 | -0.002 (-0.059 - 0.054) | 0.936 | **-0.550 (-1.046 - -0.053)** | **0.042** | -0.034 (-0.134 - 0.066) | 0.507 | 0.094 |
| Plasma levels of TMAO and its precursors were natural log-transformed. White matter hyperintensities were natural log transformed and all MRI markers were standardized to facilitate comparison. Associations are presented as adjusted mean differences (with 95% confidence interval (CI)). Model I is adjusted for age, sex, education, lipid lowering medication use, body mass index, intracranial volume and time interval to MRI. Model II is additionally adjusted for total cholesterol, HDL-cholesterol, smoking, hypertension and history of coronary heart disease. Models pertaining to white matter hyperintensities, fractional anisotropy and mean diffusivity were additionally adjusted for (normal appearing) white matter volume. Associations passing the nominal significance threshold (p<0.05) are marked bold, associations passing the FDR threshold are denoted with a *. Estimates were pooled with a random-effects meta-analysis using the inverse variance method and the DerSimonian-Laird estimator. Abbreviations: RS I-4 = Rotterdam Study cohort I, fourth visit; RS III-2 = Rotterdam Study cohort III, second visit; P-Het = P-value for heterogeneity, LLS = Leiden Longevity Study, CI = confidence interval, P-value = nominal p-value, FDR = false discovery rate. | | | | | | | | | |

**Table S1-B. Associations between plasma levels of TMAO, its precursors and brain MRI markers (model II)**

| Total brain volume (per SD) | | | | | | | | | |
| --- | --- | --- | --- | --- | --- | --- | --- | --- | --- |
|  | RS I-4 (N=898) | | RS III-2 (N=1088) | | LLS  (N=251) | | Meta-analysis (N=2237) | | |
| Model II | Mean difference (95% CI) | P-value | Mean difference (95% CI) | P-value | Mean difference (95% CI) | P-value | Mean difference (95% CI) | P-value | P-_Het_ |
| Betaine | 0.021 (-0.065 - 0.107) | 0.635 | 0.024 (-0.049 - 0.096) | 0.523 | -0.131 (-0.853 - 0.590) | 0.725 | 0.022 (-0.033 - 0.077) | 0.439 | 0.915 |
| Carnitine | -0.039 (-0.155 - 0.076) | 0.504 | 0.078 (-0.016 - 0.172) | 0.102 | 0.405 (-0.510 - 1.320) | 0.397 | 0.030 (-0.073 - 0.134) | 0.567 | 0.223 |
| Choline | **-0.118 (-0.224 - -0.012)** | **0.029** | -0.053 (-0.145 - 0.038) | 0.252 | 0.471 (-0.262 - 1.203) | 0.224 | -0.074 (-0.171 - 0.023) | 0.137 | 0.225 |
| Deoxycarnitine | -0.063 (-0.147 - 0.022) | 0.146 | 0.058 (-0.043 - 0.160) | 0.260 | 0.431 (-0.546 - 1.408) | 0.398 | -0.001 (-0.114 - 0.113) | 0.993 | 0.135 |
| TMAO | -0.021 (-0.055 - 0.013) | 0.228 | -0.009 (-0.035 - 0.018) | 0.514 | 0.201 (-0.034 - 0.435) | 0.110 | -0.010 (-0.043 - 0.022) | 0.532 | 0.174 |
| Gray matter volume (per SD) | | | | | | | | | |
|  | RS I-4 (N=898) | | RS III-2 (N=1088) | | LLS  (N=251) | | Meta-analysis (N=2237) | | |
| Model II | Mean difference (95% CI) | P-value | Mean difference (95% CI) | P-value | Mean difference (95% CI) | P-value | Mean difference (95% CI) | P-value | P-_Het_ |
| Betaine | 0.016 (-0.135 - 0.167) | 0.837 | 0.070 (-0.055 - 0.195) | 0.272 | -0.743 (-2.467 - 0.981) | 0.409 | 0.045 (-0.051 - 0.142) | 0.355 | 0.578 |
| Carnitine | **-0.232 (-0.436 - -0.029)** | **0.025** | 0.096 (-0.066 - 0.257) | 0.245 | -0.330 (-2.736 - 2.076) | 0.791 | -0.064 (-0.357 - 0.228) | 0.666 | 0.045 |
| Choline | -0.164 (-0.351 - 0.024) | 0.087 | 0.053 (-0.104 - 0.210) | 0.509 | 0.022 (-1.929 - 1.974) | 0.982 | -0.045 (-0.217 - 0.127) | 0.607 | 0.221 |
| Deoxycarnitine | -0.106 (-0.255 - 0.043) | 0.161 | 0.020 (-0.154 - 0.195) | 0.819 | 1.443 (-1.030 - 3.916) | 0.267 | -0.044 (-0.187 - 0.098) | 0.541 | 0.278 |
| TMAO | -0.043 (-0.102 - 0.017) | 0.159 | 0.035 (-0.010 - 0.080) | 0.131 | 0.470 (-0.146 - 1.086) | 0.151 | 0.007 (-0.078 - 0.092) | 0.879 | 0.040 |
| White matter volume (per SD) | | | | | | | | | |
|  | RS I-4 (N=898) | | RS III-2 (N=1088) | | LLS  (N=251) | | Meta-analysis (N=2237) | | |
| Model II | Mean difference (95% CI) | P-value | Mean difference (95% CI) | P-value | Mean difference (95% CI) | P-value | Mean difference (95% CI) | P-value | P-_Het_ |
| Betaine | 0.020 (-0.141 - 0.180) | 0.811 | -0.026 (-0.164 - 0.112) | 0.713 | -0.800 (-1.945 - 0.346) | 0.187 | -0.013 (-0.118 - 0.091) | 0.805 | 0.366 |
| Carnitine | 0.151 (-0.066 - 0.368) | 0.172 | 0.041 (-0.138 - 0.219) | 0.654 | 1.286 (-0.257 - 2.830) | 0.119 | 0.108 (-0.081 - 0.298) | 0.263 | 0.234 |
| Choline | -0.043 (-0.243 - 0.156) | 0.671 | -0.137 (-0.310 - 0.036) | 0.120 | 0.812 (-0.472 - 2.095) | 0.230 | -0.082 (-0.235 - 0.071) | 0.293 | 0.302 |
| Deoxycarnitine | -0.005 (-0.163 - 0.154) | 0.953 | 0.078 (-0.115 - 0.270) | 0.429 | 0.669 (-1.053 - 2.391) | 0.456 | 0.032 (-0.090 - 0.154) | 0.608 | 0.620 |
| TMAO | 0.005 (-0.058 - 0.069) | 0.866 | -0.047 (-0.097 - 0.003) | 0.065 | 0.183 (-0.254 - 0.621) | 0.421 | -0.023 (-0.071 - 0.025) | 0.354 | 0.283 |
| Hippocampal volume (per SD) | | | | | | | | | |
|  | RS I-4 (N=836) | | RS III-2 (N=977) | | LLS  (N=251) | | Meta-analysis (N=2064) | | |
| Model II | Mean difference (95% CI) | P-value | Mean difference (95% CI) | P-value | Mean difference (95% CI) | P-value | Mean difference (95% CI) | P-value | P-_Het_ |
| Betaine | -0.024 (-0.218 - 0.170) | 0.806 | -0.051 (-0.211 - 0.109) | 0.532 | **-1.981 (-3.407 - -0.556)** | **0.013** | -0.118 (-0.419 - 0.182) | 0.441 | 0.029 |
| Carnitine | 0.059 (-0.202 - 0.320) | 0.655 | 0.053 (-0.151 - 0.257) | 0.611 | 0.134 (-2.172 - 2.441) | 0.910 | 0.056 (-0.105 - 0.216) | 0.496 | 0.997 |
| Choline | -0.040 (-0.282 - 0.202) | 0.745 | -0.071 (-0.269 - 0.127) | 0.481 | -0.453 (-2.310 - 1.404) | 0.638 | -0.061 (-0.214 - 0.091) | 0.432 | 0.900 |
| Deoxycarnitine | -0.064 (-0.251 - 0.124) | 0.504 | 0.017 (-0.203 - 0.237) | 0.882 | 1.189 (-1.199 - 3.578) | 0.341 | -0.025 (-0.168 - 0.117) | 0.728 | 0.522 |
| TMAO | 0.000 (-0.077 - 0.077) | 0.999 | 0.003 (-0.053 - 0.058) | 0.929 | 0.250 (-0.363 - 0.864) | 0.433 | 0.003 (-0.041 - 0.048) | 0.885 | 0.729 |
| White matter hyperintensities (per SD) | | | | | | | | | |
|  | RS I-4 (N=898) | | RS III-2 (N=1088) | | LLS  (N=232) | | Meta-analysis (N=2218) | | |
| Model II | Mean difference (95% CI) | P-value | Mean difference (95% CI) | P-value | Mean difference (95% CI) | P-value | Mean difference (95% CI) | P-value | P-_Het_ |
| Betaine | 0.046 (-0.171 - 0.262) | 0.680 | 0.037 (-0.125 - 0.199) | 0.655 | 0.345 (-1.675 - 2.365) | 0.742 | 0.041 (-0.089 - 0.172) | 0.532 | 0.955 |
| Carnitine | -0.083 (-0.376 - 0.210) | 0.579 | **-0.307 (-0.517 - -0.098)** | **0.004*** | -0.511 (-3.305 - 2.284) | 0.725 | **-0.232 (-0.402 - -0.062)** | **0.008*** | 0.465 |
| Choline | 0.204 (-0.065 - 0.473) | 0.137 | **0.209 ( 0.005 - 0.413)** | **0.044** | -0.637 (-2.677 - 1.403) | 0.550 | **0.202 ( 0.040 - 0.364)** | **0.015*** | 0.721 |
| Deoxycarnitine | 0.121 (-0.093 - 0.334) | 0.268 | 0.118 (-0.108 - 0.345) | 0.306 | -0.349 (-2.913 - 2.214) | 0.793 | 0.118 (-0.038 - 0.273) | 0.137 | 0.938 |
| TMAO | 0.005 (-0.081 - 0.090) | 0.913 | 0.020 (-0.039 - 0.079) | 0.503 | -0.132 (-0.815 - 0.551) | 0.710 | 0.014 (-0.034 - 0.063) | 0.558 | 0.879 |
| Fractional anisotropy (per SD) | | | | | | | | | |
|  | RS I-4 (N=728) | | RS III-2 (N=1062) | | LLS  (N=230) | | Meta-analysis (N=2020) | | |
| Model II | Mean difference (95% CI) | P-value | Mean difference (95% CI) | P-value | Mean difference (95% CI) | P-value | Mean difference (95% CI) | P-value | P-_Het_ |
| Betaine | -0.187 (-0.476 - 0.102) | 0.205 | -0.159 (-0.365 - 0.047) | 0.130 | 0.674 (-1.473 - 2.822) | 0.550 | -0.163 (-0.330 - 0.004) | 0.055 | 0.736 |
| Carnitine | -0.147 (-0.531 - 0.237) | 0.452 | 0.171 (-0.095 - 0.437) | 0.209 | -1.698 (-4.693 - 1.297) | 0.288 | 0.017 (-0.313 - 0.348) | 0.918 | 0.211 |
| Choline | -0.243 (-0.595 - 0.109) | 0.176 | -0.134 (-0.392 - 0.124) | 0.309 | -0.187 (-2.492 - 2.118) | 0.876 | -0.173 (-0.380 - 0.035) | 0.103 | 0.887 |
| Deoxycarnitine | 0.155 (-0.114 - 0.423) | 0.258 | -0.025 (-0.313 - 0.262) | 0.863 | -0.130 (-2.932 - 2.673) | 0.929 | 0.070 (-0.126 - 0.265) | 0.484 | 0.661 |
| TMAO | 0.092 (-0.022 - 0.205) | 0.112 | -0.034 (-0.109 - 0.041) | 0.370 | 0.290 (-0.406 - 0.986) | 0.430 | 0.027 (-0.086 - 0.140) | 0.638 | 0.139 |
| Mean diffusivity (per SD) | | | | | | | | | |
|  | RS I-4 (N=728) | | RS III-2 (N=1062) | | LLS  (N=230) | | Meta-analysis (N=x) | | |
| Model II | Mean difference (95% CI) | P-value | Mean difference (95% CI) | P-value | Mean difference (95% CI) | P-value | Mean difference (95% CI) | P-value | P-_Het_ |
| Betaine | -0.008 (-0.250 - 0.234) | 0.950 | -0.010 (-0.165 - 0.146) | 0.903 | -0.098 (-1.782 - 1.585) | 0.911 | -0.010 (-0.140 - 0.120) | 0.881 | 0.995 |
| Carnitine | 0.083 (-0.238 - 0.404) | 0.612 | **-0.236 (-0.436 - -0.035)** | **0.021** | -0.851 (-3.818 - 2.115) | 0.584 | -0.118 (-0.374 - 0.138) | 0.368 | 0.227 |
| Choline | 0.020 (-0.275 - 0.315) | 0.894 | -0.026 (-0.221 - 0.169) | 0.797 | -1.074 (-2.885 - 0.737) | 0.268 | -0.020 (-0.182 - 0.141) | 0.804 | 0.502 |
| Deoxycarnitine | -0.152 (-0.376 - 0.072) | 0.183 | -0.023 (-0.239 - 0.194) | 0.838 | -1.154 (-3.254 - 0.946) | 0.303 | -0.092 (-0.247 - 0.064) | 0.248 | 0.439 |
| TMAO | -0.035 (-0.129 - 0.060) | 0.473 | -0.005 (-0.062 - 0.051) | 0.862 | -0.391 (-0.947 - 0.165) | 0.193 | -0.016 (-0.067 - 0.034) | 0.522 | 0.359 |
| Plasma levels of TMAO and its precursors were natural log-transformed. White matter hyperintensities were natural log transformed and all MRI markers were standardized to facilitate comparison. Associations are presented as adjusted mean differences (with 95% confidence interval (CI)). Model I is adjusted for age, sex, education, lipid lowering medication use, body mass index, intracranial volume and time interval to MRI. Model II is additionally adjusted for total cholesterol, HDL-cholesterol, smoking, hypertension and history of coronary heart disease. Models pertaining to white matter hyperintensities, fractional anisotropy and mean diffusivity were additionally adjusted for (normal appearing) white matter volume. Associations passing the nominal significance threshold (p<0.05) are marked bold, associations passing the FDR threshold are denoted with a *. Estimates were pooled with a random-effects meta-analysis using the inverse variance method and the DerSimonian-Laird estimator. Abbreviations: RS I-4 = Rotterdam Study cohort I, fourth visit; RS III-2 = Rotterdam Study cohort III, second visit; LLS = Leiden Longevity Study, P-Het = P-value for heterogeneity, CI = confidence interval, P-value = nominal p-value, FDR = false discovery rate. | | | | | | | | | |

**Table S2: Associations between plasma levels of TMAO, its precursors and incident dementia with impaired renal function**

|  | Incident dementia | | | Incident Alzheimer’s dementia. | | |
| --- | --- | --- | --- | --- | --- | --- |
| Model I | n/N | HR (95% CI) | P-value | n/N | HR (95% CI) | P-value |
| Betaine | 63/245 | 1.27 (0.46 - 3.53) | 0.64 | 51/245 | 1.07 (0.35 - 3.25) | 0.90 |
| Carnitine | 63/245 | 1.64 (0.41 - 6.54) | 0.48 | 51/245 | 1.68 (0.37 - 7.64) | 0.50 |
| Choline | 63/245 | 2.12 (0.63 - 7.18) | 0.23 | 51/245 | 1.40 (0.37 - 5.24) | 0.62 |
| Deoxycarnitine | 63/245 | 1.64 (0.46 - 5.79) | 0.44 | 51/245 | 0.84 (0.20 - 3.57) | 0.81 |
| TMAO | 63/245 | **1.73 (1.16 - 2.60)** | **0.01*** | 51/245 | **1.79 (1.15 - 2.80)** | **0.01** |
| Model II | n/N | HR (95% CI) | P-value | n/N | HR (95% CI) | P-value |
| Betaine | 62/238 | 1.54 (0.53 - 4.46) | 0.43 | 50/238 | 1.45 (0.45 - 4.73) | 0.53 |
| Carnitine | 62/238 | 1.35 (0.34 - 5.37) | 0.67 | 50/238 | 1.37 (0.29 - 6.39) | 0.69 |
| Choline | 62/238 | 2.03 (0.60 - 6.91) | 0.25 | 50/238 | 1.34 (0.35 - 5.11) | 0.67 |
| Deoxycarnitine | 62/238 | 1.35 (0.36 - 5.13) | 0.66 | 50/238 | 0.58 (0.12 - 2.82) | 0.50 |
| TMAO | 62/238 | **1.69 (1.12 - 2.56)** | **0.01** | 50/238 | **1.81 (1.15 - 2.84)** | **0.01** |
| Impaired renal function was defined as an estimated glomerular filtration rate (eGFR) <60 mL/kg (n=245). Note that models pertaining to incident dementia only include participants from RS I-4 = Rotterdam Study cohort I, fourth visit. Plasma levels of TMAO and its precursors were natural log-transformed. Associations with incident dementia are presented as hazard ratios (HR), with 95% confidence interval (CI). Model I is adjusted for age, sex, education, lipid lowering medication use and body mass index. Model II is additionally adjusted for total cholesterol, HDL-cholesterol, smoking, hypertension and history of coronary heart disease. Associations passing the nominal significance threshold (p<0.05) are marked bold, associations passing the FDR threshold are denoted with a *. Abbreviations: P-Het = P-value for heterogeneity, CI = confidence interval, P-value = nominal p-value, FDR = false discovery rate, n/N = number of incident cases/number of included participants, HR = hazard ratio. | | | | | | |

.

**Figure S3: Associations between TMAO, its precursors and incident dementia, as well as Alzheimer’s disease, stratified by sex**

**Figure S3:** Associations between TMAO, its precursors and incident dementia, as well as Alzheimer’s disease, stratified by sex. Among 1457 females, 334 developed incident (all-cause) dementia, from which 271 individuals had Alzheimer’s disease. Among 1060 males, 179 developed incident (all-cause) dementia, from which 131 individuals had Alzheimer’s disease. Effect estimates from Cox proportional hazard models are presented as hazard ratios with 95% confidence intervals for model II.

**Figure S4: Correlation between TMAO and its precursors in plasma**

**Figure S4:** Correlation plot displaying the Pearsons’ correlation between TMAO and its precursors. Positive correlations are denoted in blue, whereas negative correlations are displayed in red.

**Figure S5: Scatterplots of plasma TMAO and its precursors and neuroimaging markers**
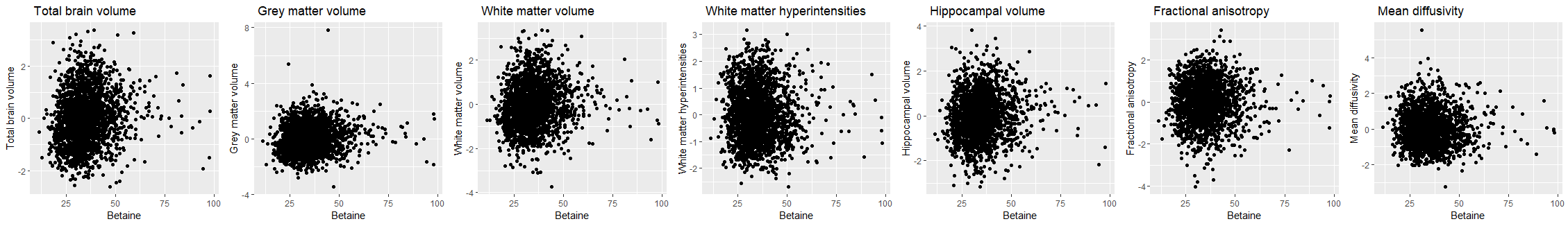


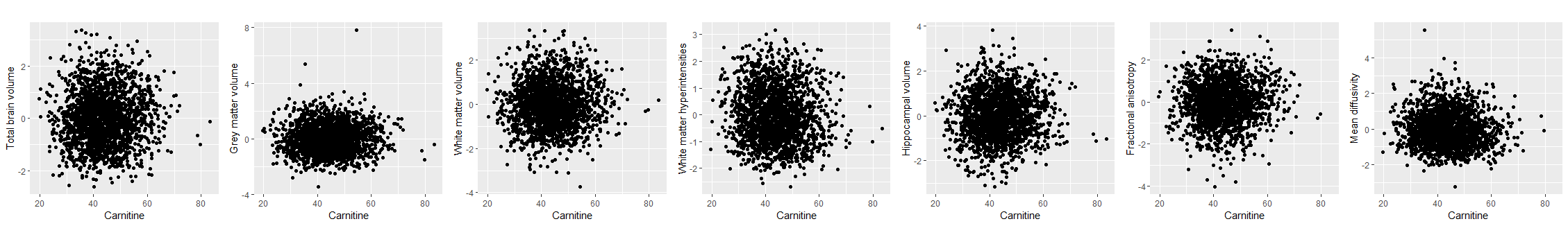


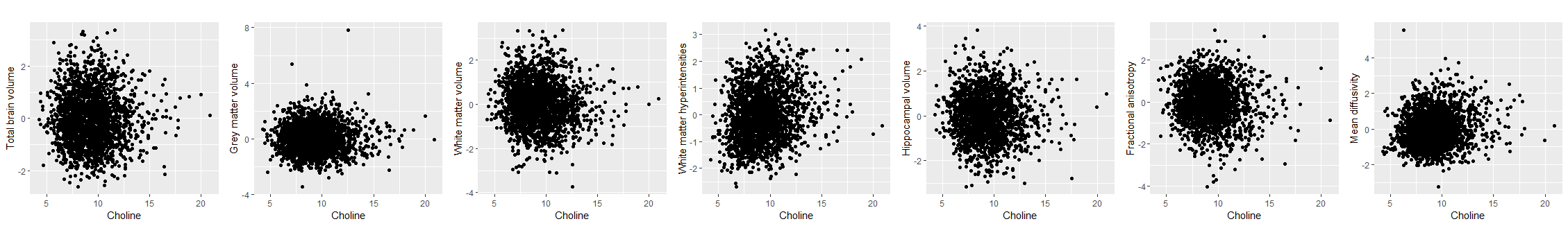


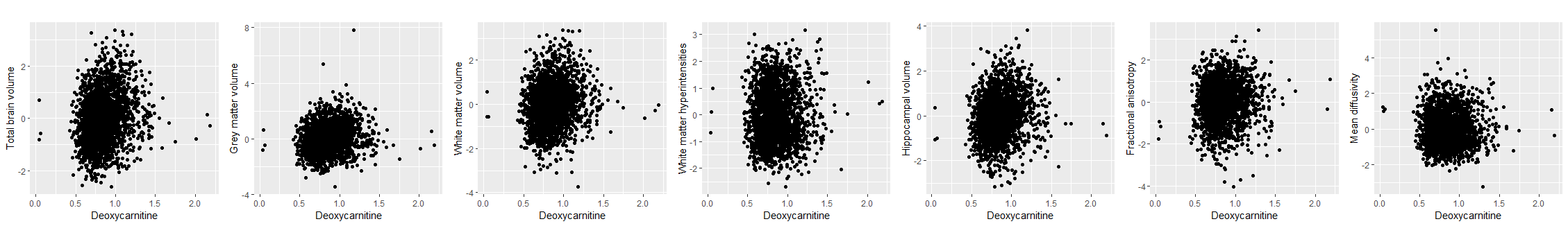


Before log-tranformation: TMAO


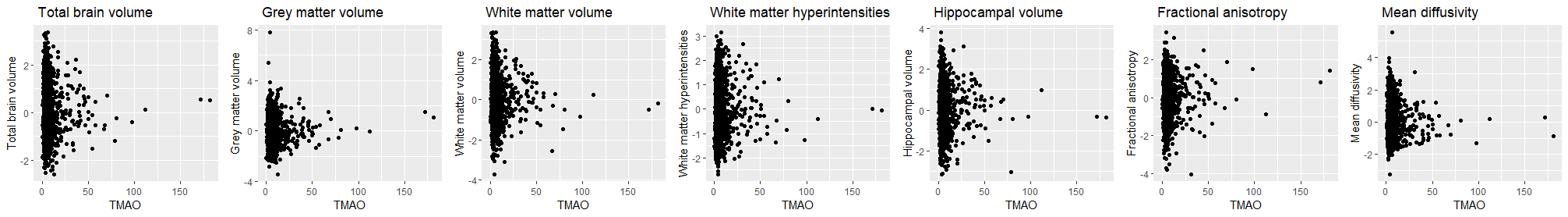


After log-tranformation: TMAO


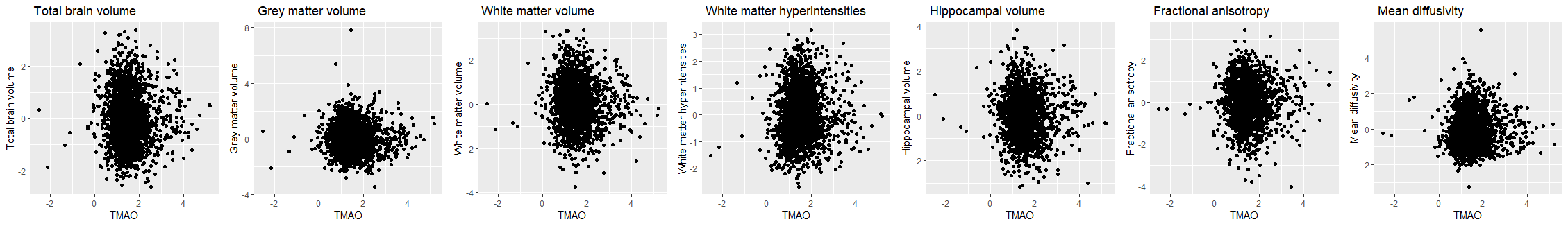


**Figure S5:** Scatterplots depicting the relationship between plasma TMAO, its precursors, and neuroimaging markers. Although outliers were observed for several gut-related metabolites, those associated with TMAO were evident. To ensure the data followed a normal distribution for linear regression models, a log-transformation was performed on all gut-related metabolites and the volume of white matter hyperintensities. An example of this transformation is illustrated above for TMAO. All neuroimaging markers are presented standardized to facilitate comparison.
